# The therapeutic effect of capsaicin on oropharyngeal dysphagia: A systematic review and meta-analysis

**DOI:** 10.3389/fnagi.2022.931016

**Published:** 2022-11-08

**Authors:** Cong-wen Yang, Ru-dong Chen, Meng-ting Feng, Meng-zhen Zhang, Wei Liu, Xu-chang Liu, Da-chuan Wang

**Affiliations:** ^1^Department of Neurosurgery, Affiliated Hospital of Weifang Medical University, Weifang, China; ^2^Department of Spinal Surgery, Provincial Hospital Affiliated to Shandong First Medical University, Jinan, China; ^3^Department of Pediatrics, Jilin University, Changchun, China; ^4^Preventive Medicine, Qingdao University, Qingdao, China

**Keywords:** oropharyngeal dysphagia, capsaicin, TRPV1, randomized controlled trials, swallowing function score

## Abstract

**Objectives:**

Capsaicin is a specific agonist of TRPV1 (multimodal sensory receptor), which improves oropharyngeal dysphagia by increasing sensory input from the oropharynx and hypopharynx and by increasing repetitive stimulation of the cerebral cortex. The aim of this systematic review was to evaluate the therapeutic effect of capsaicin on swallowing disorders in stroke patients and the elderly.

**Method:**

We searched Medline, Embase, PubMed, and Cochrane Library databases. We used the Mesh terms search database to screen all clinical trials that complied with the inclusion criteria. Studies were subjected to literature screening, quality assessment, and data extraction to remove studies that did not meet the inclusion criteria. After literature screening, quality assessment, and data extraction, a systematic review and meta-analysis of the included study were performed.

**Results:**

This systematic review and meta-analysis were prospectively registered on PROSPERO under registration number CRD42022313958. Five high-quality randomized controlled trials were ultimately included. The results of our meta-analysis showed a more significant reduction in swallowing function score change in the capsaicin group compared to the control group [SMD = −1.30, 95% CI: (−2.35, −0.25), *P* = 0.01] and on the Water swallowing test the improvement was significantly higher in the capsaicin group [RR = 2.46, 95% CI: (1.73, 3.50), *P* < 0.0001].

**Conclusions:**

Although the results of our meta-analysis showed that capsaicin improved swallowing function, most studies had an unclear bias and included few studies. More studies are needed to support this in the future.

**Systematic review registration:**

www.crd.york.ac.uk/prospero/display_record.php?RecordID=304061, identifier: 304061.

## Introduction

Oropharyngeal dysphagia (OD) is caused by a variety of diseases, the most common causes being stroke and aging (Baijens et al., 2016). As the population is rapidly aging, stroke has gradually become the leading cause of death in the elderly (Lui and Nguyen, 2018). OD is a common complication after acute stroke. It is estimated that 37–78% of stroke survivors suffer from dysphagia (Marin et al., 2018). Moreover, in a survey of older adults, 20–23% of them had swallowing disorders (Serra-Prat et al., 2011). Patients with mild dysphagia may experience choking on water, difficulty in eating, and recurrent fever (Cui et al., 2020). Severely patients are prone to aspiration pneumonia and asphyxia and malnutrition, which seriously impair the quality of life of patients and sometimes even endanger their lives (Goldsmith et al., 2021). Traditional treatments for dysphagia include dietary modification, postural modification, motor training, and swallowing motions (Speyer et al., 2010). Swallowing training is a cumbersome procedure with a long duration of treatment and negative impact on patient compliance (White et al., 2008). Therefore, there is a need for continued research into new treatment methods.

The pathological condition of swallowing can be divided into three stages: the oral stage, the pharyngeal stage, and the esophageal phase (Sasegbon and Hamdy, 2017). Except for the oral phase, swallowing movements are involuntary and are accomplished mainly by reflexes (Hashimoto et al., 2021). Studies have shown that sensory input is important for the initiation and regulation of swallowing (Steele and Miller, 2010), and the cause of the patient's swallowing dysfunction may be a disruption in the connection between cortical sensory afferents and the brainstem (Sasegbon and Hamdy, 2021). Those aging or stroke patients have lengthened latency and reduced amplitude of the characteristic peaks of pharyngeal sensory evoked potentials (PSEP) (Cabib et al., 2017), in addition to symmetric PSEP and its cortical representation loss in patients with chronic dysphagia after stroke (Cabib et al., 2020a). Eventually, impaired conduction and integration of sensory input may affect output pathways, leading to impaired oropharyngeal responses (Cabib et al., 2020b).

A multimodal sensory receptor TRPV1 was found to be expressed in epithelial cells and sensory neurons of the population pharynx and larynx (Alvarez-Berdugo et al., 2016), which induces peripheral sensory neurons, modulates sensory input from oropharyngeal and hypopharyngeal regions and improves swallowing function (Steele and Miller, 2010). TRPV1 has two effects on improving swallowing ability (Rofes et al., 2013). It increases sensory input from the oropharynx and hypopharynx and increases repetitive stimulation of the cerebral cortex (Cabib et al., 2016). In addition to oral pathway stimulation, there are also nasal and ear pathways that may stimulate the swallowing reflex through TRPV1 receptors (Ravindran et al., 2016). Capsaicin is a specific agonist of TRPV1 (Ferreira et al., 2020). Several studies have shown that TRPV1 stimulation by capsaicin increased the concentration of salivary substance P (SP) (Kondo et al., 2017; Cui et al., 2020). SP is a neuropeptide that induces centrally originated swallowing through afferent stimulation of the vagus and pharyngeal nerves and enhances swallowing and cough reflexes (Mazzone and Undem, 2016). In addition, capsaicin shortens laryngeal vestibule closure (LVC) and upper esophageal sphincter opening (UESO) to improves swallowing safety (Rofes et al., 2013). Therefore, this systematic review attempts to assess the therapeutic effect of capsaicin on dysphagia in stroke patients and the elderly, providing new evidence for the rehabilitation of patients with dysphagia.

## Materials and methods

The protocol for this review was registered PROSPERO (International prospective register of systematic reviews) and the registration number was CRD42022313958 (Supplementary material 1).

### Search strategy

The databases searched for this systematic review and meta-analysis included Medline, Embase, PubMed, and the Cochrane Library. The search keywords used MeSH terms which were “swallowing” OR “swallowing disorders” OR “deglutition disorders” OR “dysphagia” AND “capsaicin” OR “TRP” OR “vanilloid receptor agonist” (the search strategy is presented in Supplementary material 2). Randomized controlled trials (RCTs) published up to July 2022 were included based on inclusion and exclusion criteria. Two researchers with medical specialties independently assessed the title and abstract of each item using predetermined inclusion and exclusion criteria. When judgment could not be made on the basis of titles and abstracts, the full text of the articles was obtained. There was no potential bias between the two researchers and agreement was reached through discussion.

### Inclusion and exclusion criteria

#### Participants/population

Inclusion criteria: (1) patients with dysphagia associated with aging and stroke [Elderly or stroke patients were evaluated for Oropharyngeal dysphagia by Standardized Swallowing Assessment (SSA) scales or Volume-Viscosity Swallowing Test (V-VST) or Video Fluoroscopic Swallowing (VFS)]; (2) age > 18 years old; (3) patients were willing to participant the study and signed the informed consent.

Exclusion criteria: (1) patients with lung disease and heart disease who cannot receive stimulation; (2) patient suffers from other diseases that may affect swallowing function; (3) patients who were allergic to capsaicin.

#### Intervention(s), exposure(s)

Inclusion criteria: (1) patients received oral or topical capsaicin stimulation; (2) may be combined with other treatments (ice stimulation, swallowing training).

#### Comparator(s)/control

Patients in the control group were treated with placebo or combined with other drugs same as in the experimental group. No control group was set and studies comparing capsaicin with other treatments were excluded.

#### Types of study to be included

The inclusion criteria were (1) only published RCTs were included; (2) studies with a follow-up of at least 80% and at least one primary outcome and (3) those with complete treatment outcomes.

Exclusion criteria: review articles, animal studies, a case study, not relevant to the question and data was not extractable.

### Outcomes

The primary outcomes included: swallowing function score and water swallowing test. Secondary outcomes: efficacy of capsaicin, substance P (SP) concentrations, individual latency time, the standardized low-resolution brain electromagnetic tomography software (sLORETA), and safety of the treatment.

### Data extraction

Two researchers individually extracted and tabulated data on authors, date of publication, study site, sample size, sample characteristics, gender, age, experimental design, interventions, mode of administration, and outcomes. For incomplete data, the authors were contacted and any missing data were obtained. Controversial data were discussed or a third investigator was consulted to resolve any disagreements.

### Risk of bias and quality assessment

The PRISMA guidelines (Moher et al., 2009) and Cochrane Handbook (Higgins et al., 2019) were used to evaluate the quality of the results of all included studies to make sure that the results of our meta-analysis were reliable and authentic (Moher et al., 2009). The PRISMA 2020 checklist (Page et al., 2021) was shown in Supplementary Table 1.

**Figure 1:**
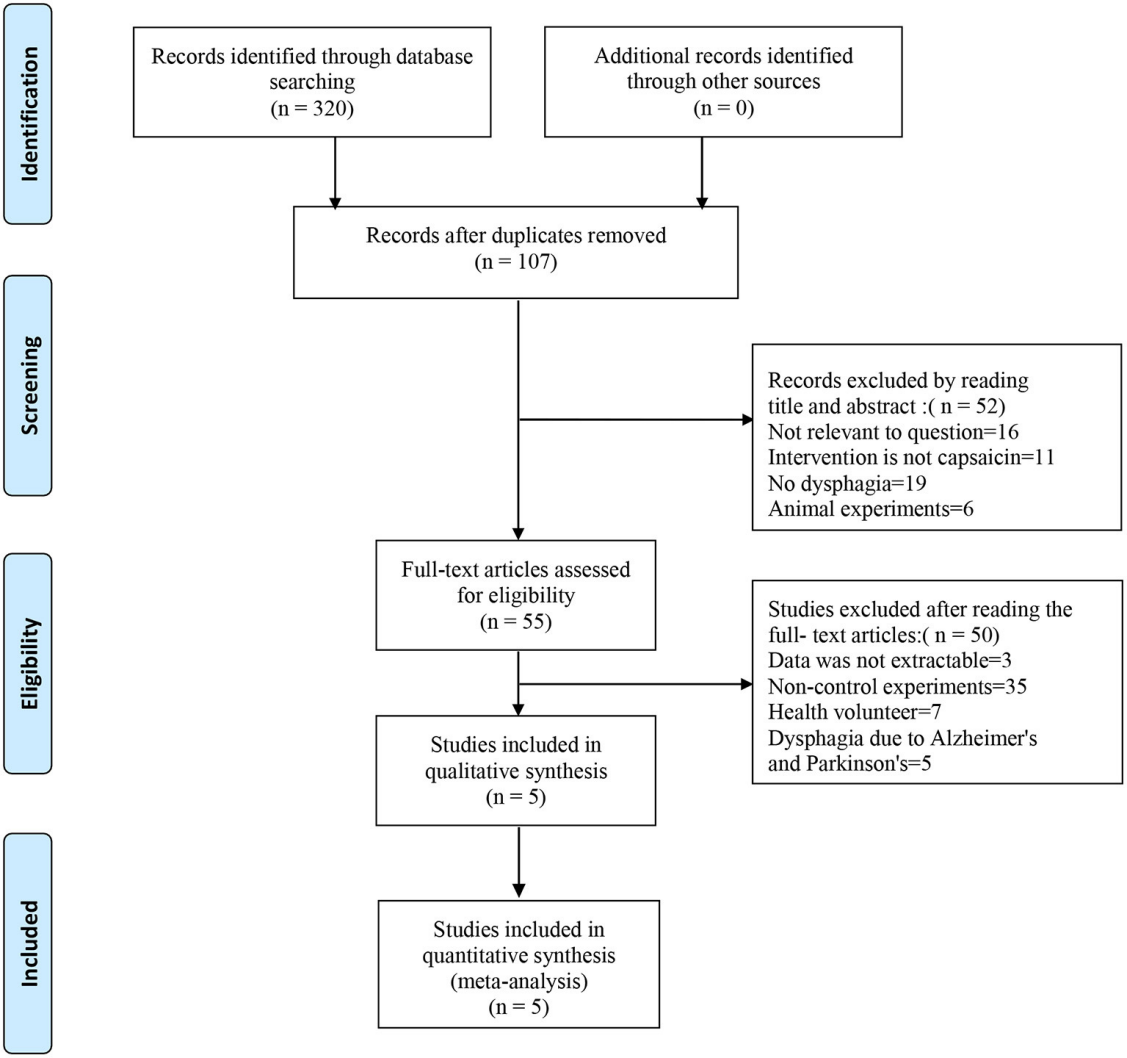
Flowchart of study selection.

**Figure 2:**
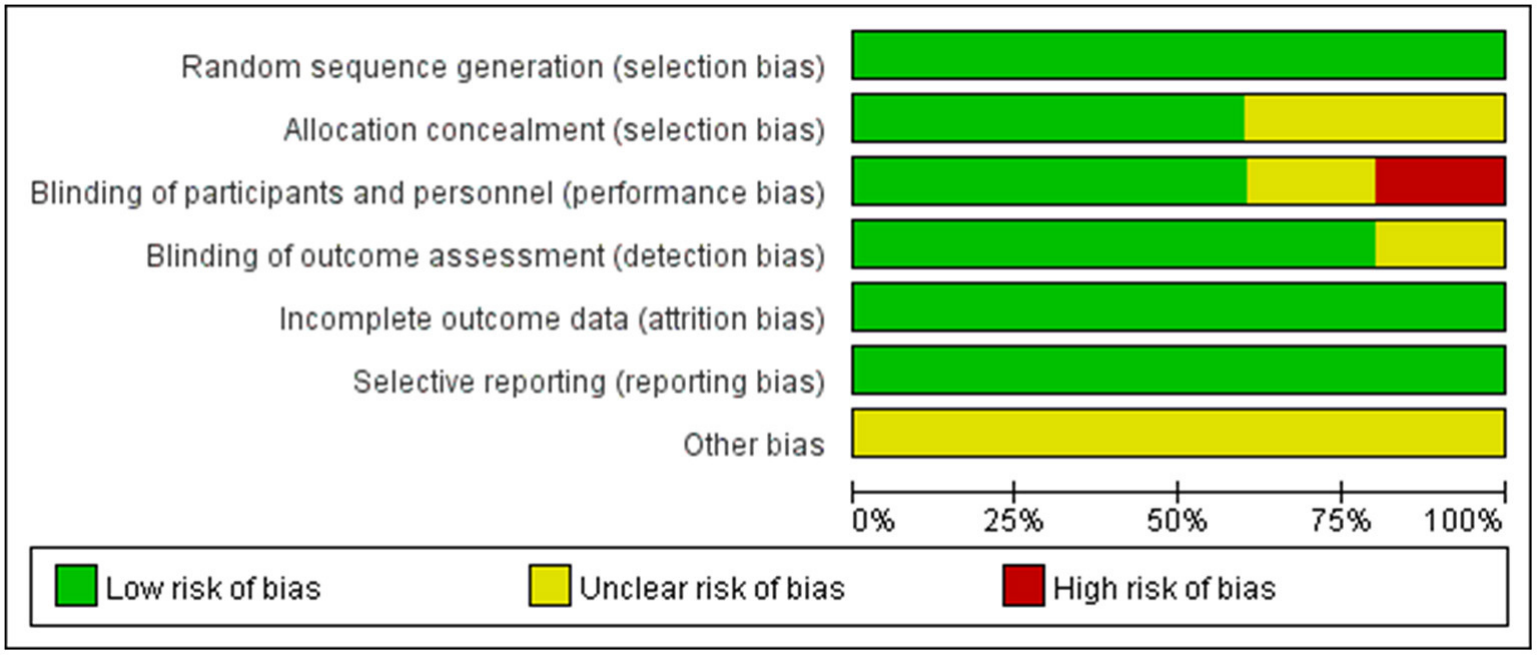
Risk of bias graph.

According to the Cochrane Handbook for Systematic Reviews of Interventions, the methodological quality and basis of the included RCTs were assessed as follows: randomization, allocation concealment, blind method, selective reporting, incomplete outcome data, and other bias (Faggion, 2015) (Figures 1, 2).

### Statistical analysis and assessment of publication bias

Review Manager Software (Version 5.3) was used to perform the meta-analyses. For heterogeneity analysis, a fixed-effects model was used when I^2^ < 50% and *P* > 0.1 (Higgins et al., 2009). The meta-analysis was otherwise performed by applying a random-effects model (Friedrich et al., 2011). For dichotomous outcomes, the results were presented as relative risks (RR) with a 95% CI (Thakkinstian et al., 2005). The Chi-square test was used to evaluate the heterogeneity of studies based on the values of P and I^2^ (Shi et al., 2020). Mean difference (MD) or standardized mean difference (SMD) was used to assess continuous outcomes with 95% confidence intervals (CI) (Deeks et al., 2019). Subgroup analyses and random-effects models were performed to reduce heterogeneity (Langan, 2022), but potential heterogeneity remains unavoidable.

Some studies reported the median, first and third quartiles, and maximum and minimum values. To perform a valid meta-analysis of continuous variables, these data were then transformed into means and standard deviations using the Box-Cox transformation method (Langan, 2022). Change in swallowing scores before and after the intervention was calculated using a statistical formula (x̄= x̄_1_- x̄_2_; S= $\sqrt{{S1}^{2}+{S2}^{2}}$, x̄, Mean; S, Standard deviation).

Qualitative assessment of the funnel plot to determine publication bias, and visual inspection to determine whether there are any asymmetries (Sterne et al., 2011). Making funnel plots with Review Manager Software.

## Results

### Search results

**Figure 3:**
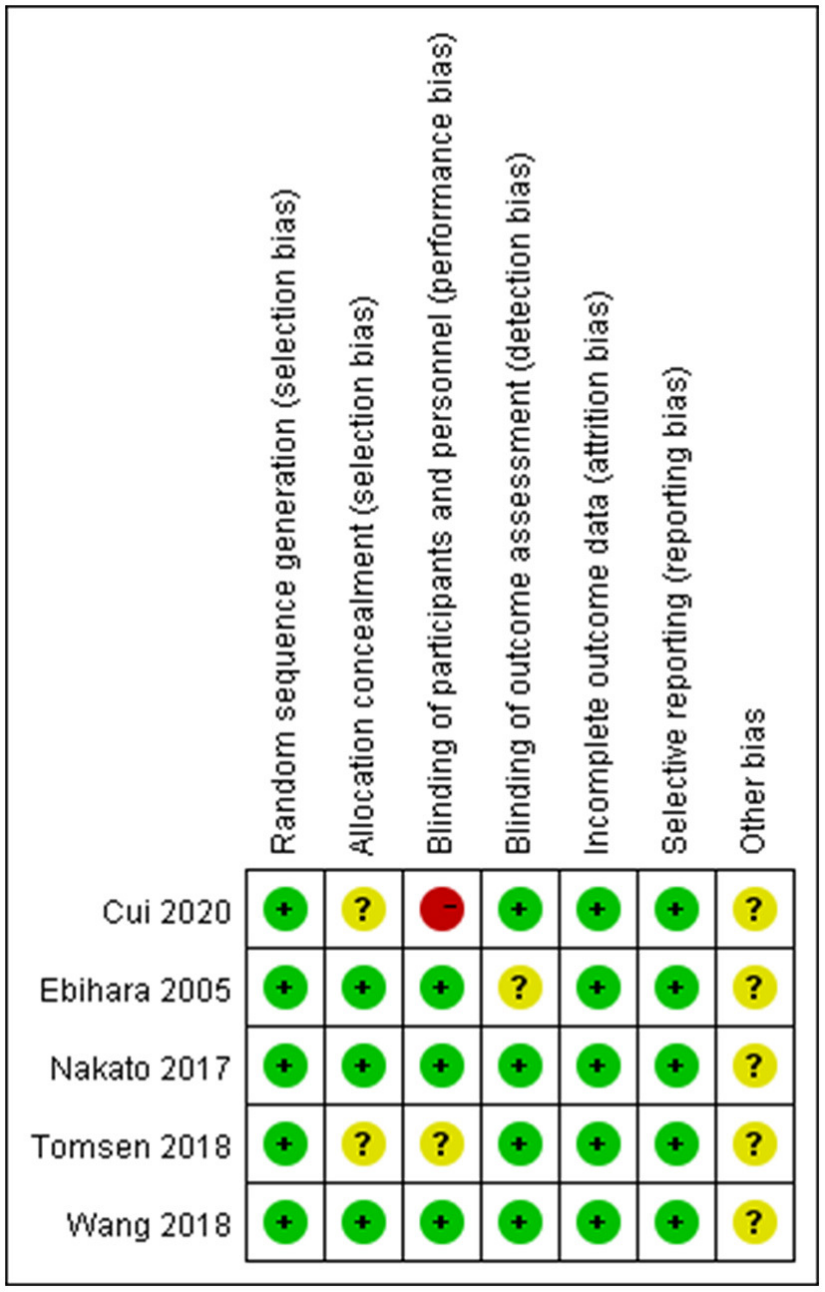
Risk of bias summary.

The search strategies are presented in Figure 3. We searched Medline, Embase, PubMed, and the Cochrane Library databases. Three hundred twenty studies were initially identified, and 107 studies were retained after excluding duplicate records. Reading the titles and abstracts, 52 studies were excluded that did not conform to the purpose of the study, such as excluding dysphagia and capsaicin. After careful reading of the full text of 55 studies, 50 studies that did not meet the inclusion criteria were excluded. Five studies were eventually included in this systematic review (Ebihara et al., 2005; Kondo et al., 2017; Nakato et al., 2017; Tomsen et al., 2019; Wang et al., 2019; Cui et al., 2020). Table 1 describes the study design.

**Table 1:** Study design.

**References**	**Swallowing problems and etiology**	**Intervention/ control**	**Drug delivery method**	**Observed duration**
Cui et al. (2020)	Stroke	Capsaicin and ice stimulation/ice stimulation	Stimulate the soft palate, palatal arch, posterior pharyngeal wall, and posterior tongue in turn with a cotton swab	21d
Wang et al. (2019)	Stroke	Capsaicin/Placebo	A cotton swab soaked in the capsaicin solution at a cold (4°C) temperature was used to dab the oropharyngeal mucosa region in the thermal tactile stimulation.	21d
Nakato et al. (2017)*	Older patients with oropharyngeal dysphagia (age ≥ 55 years).	Capsaicin plus^®^/Placebo	Oral	14d
Ebihara et al. (2005)	Elderly people who have suffered from cerebrovascular disease.	Capsaicin/Placebo	Oral	28d
Tomsen et al. (2019)	Older patients (>70 years) with OD associated with aging.	TRPV1 agonist (capsaicinoids sauce)/Placebo	Oropharyngeal sensory stimulation	10d

* A crossover randomized trial; RCT, Randomized controlled trial.

### Primary outcomes

#### Swallowing function score change

**Figure 4:**
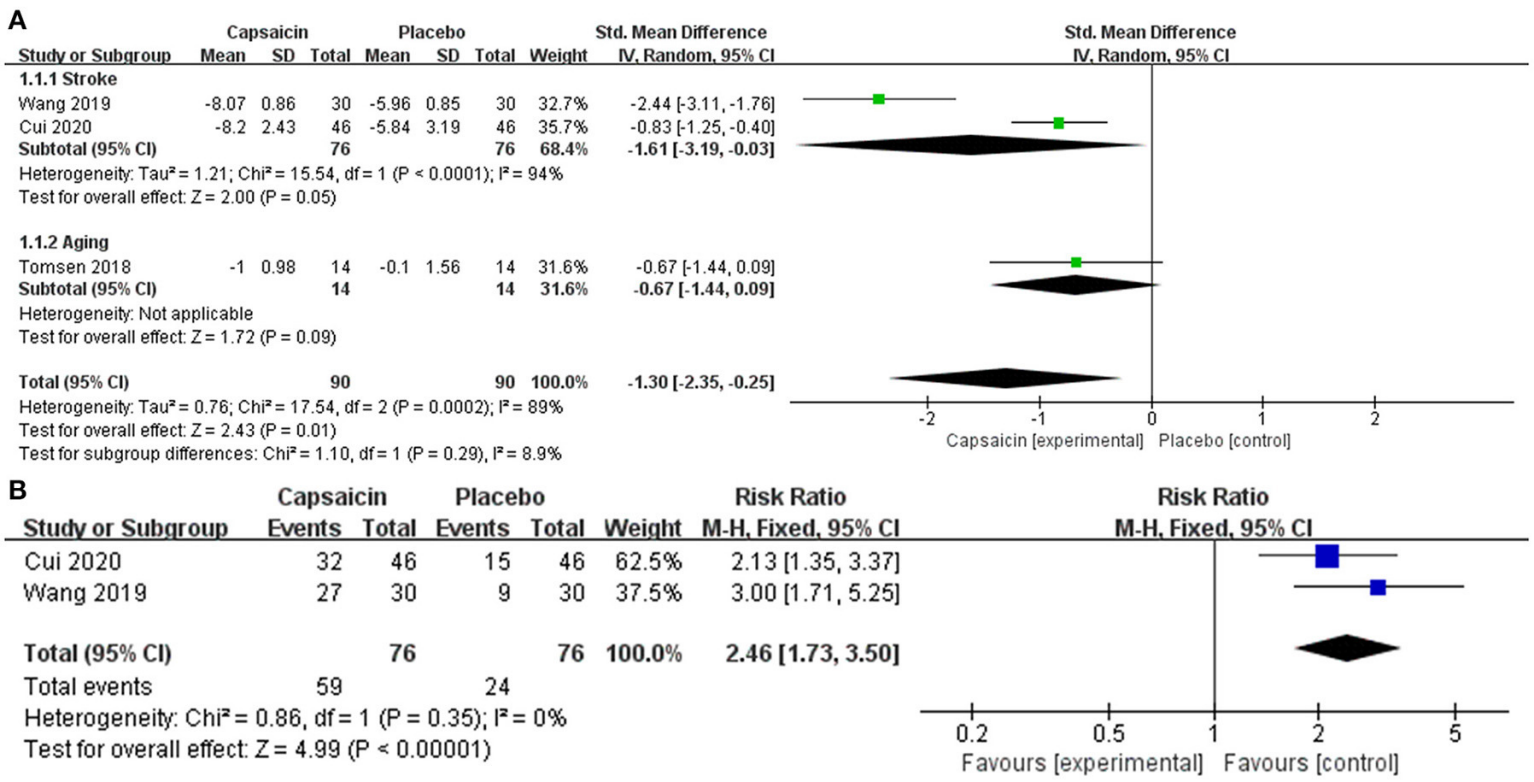
Forest plot demonstrates the effectiveness of capsaicin for oropharyngeal dysphagia: **(A)** swallowing function, **(B)** water swallowing test.

Studies Cui et al., Wang et al., and Tomsen et al. assessed swallowing function, using the SSA, V-VST and VFS scoring tools, respectively. Subgroup analysis was used to evaluate the effect of capsaicin on swallowing function scores. The meta-analysis results showed a significant difference between the capsaicin and control groups [SMD = −1.30, 95% CI: (−2.35, −0.25), *P* = 0.01; Figure 4A]. Therefore, the change in olfactory scores was more pronounced with capsaicin compared to the control group. It can be shown that capsaicin has an improving effect on swallowing function.

**Figure 5:**
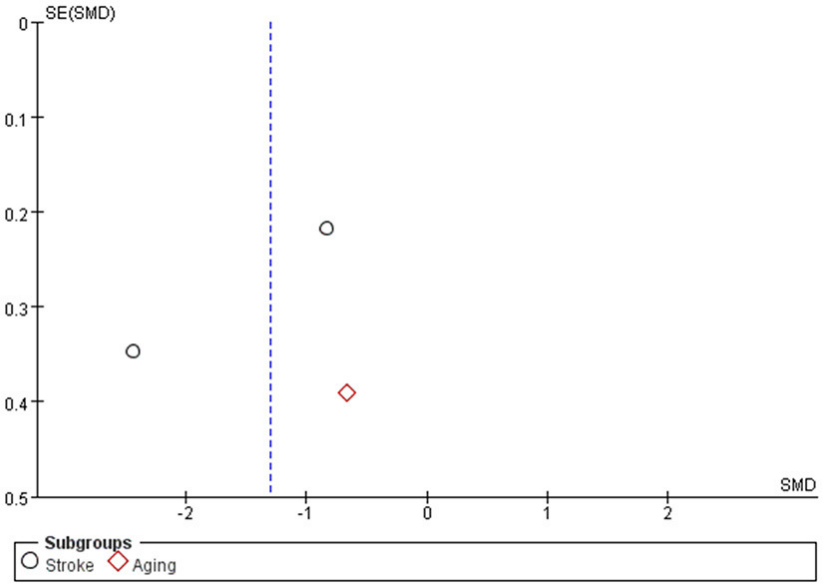
Funnel plot of swallowing function.

The funnel plot (Figure 5) is symmetrical showed a low risk of publication bias.

#### Water swallowing test

A meta-analysis of the treatment efficacy of the water swallowing test after administration of capsaicin or placebo. Treatment is considered effective when the experimental grade is raised to level I or II. The heterogeneity was observed in forest plots (I^2^ = 0%, *p* = 0.35). There is no heterogeneity therefore a fixed-effects model is used. Relative risks (RR) with 95% confidence intervals (CI) were used to assess the results of the dichotomous method. This meta-analysis results showed a significant difference between the capsaicin and control groups [RR = 2.46, 95% CI: (1.73, 3.50), *P* < 0.0001; Figure 4B].

### Second outcome

#### Efficacy of capsaicin

Treatment was effective in a significantly higher number of patients (38.8%) in the capsaicin group than those who took the placebo (6.1%, *p* < 0.001) in Nakato's study (Nakato et al., 2017).

#### Substance P concentrations

In Cui's study, the serum substance P level in the capsaicin group was significantly higher than in the control group (*p* = 0.007) (Cui et al., 2020). Furthermore, in Nakato's study, the administration of capsaicin caused a reduction in the cervical esophageal wall opening time along with an increase in SP in saliva (*p* = 0.001) (Nakato et al., 2017).

#### Individual latency time

In Ebihara's study, individual latency time of the swallowing reflex was significantly better in the capsaicin group than before the start of the study (*p* < 0.05) (Ebihara et al., 2005).

#### The standardized low-resolution brain electromagnetic tomography software (sLORETA)

In Tomsen's study, measurement results with sLORETA show that compared with basal activation, patients that received the capsaicin treatment showed a significant reduction in cortical activity at the N1, P1 and N2 peaks (*p* = 0.0002) (Tomsen et al., 2019). And patients in the capsaicin group had a significant reduction in laryngeal vestibule closure time compared to the control group. Studies show that impaired safety of deglutition and aspirations in older people are mainly caused by delayed LVC (Tomsen et al., 2019).

#### Safety of the treatment

In terms of treatment safety, only two adverse events for respiratory infections were reported in Wang's study (Wang et al., 2019), one in the capsaicin intervention group and one in the placebo control group. No treatment-related adverse events were identified in the other five studies.

## Discussion

The six randomized controlled trials included in this study were high-quality studies by quality assessment. The overall risk of bias in this study was low level.

The results of this meta showed that swallowing scores improved more in the capsaicin group compared to the control group in stroke patients and elderly patients. In our meta-analysis of the water swallowing experiment, the increased grade of the patients was considered an improvement in swallowing function. The grade was significantly higher in the capsaicin group compared to the control group (RR = 2.46, *P* < 0.0001). Therefore, both meta-analyses demonstrated that capsaicin can improve swallowing function. The Swallowing Function Test describes basic aspects of dysphagia, but it is not the gold standard for evaluating dysphagia, and clinical outcomes may be influenced by other important parameters, such as consciousness, cognitive ability, and language impairment, which can have an impact on swallowing scores. Therefore, caution is needed when using swallowing function tests to describe swallowing function.

In addition, capsaicin can mediate the local release of substance P (SP). Two studies reported the concentration of substance p in patients' serum and saliva, respectively. The SP of the capsaicin group was significantly higher than that of the control group in both studies.

Stimulation of transient receptor potentials has been reported as a target for the treatment of swallowing disorders, and activation of transient receptor potentials may improve swallowing function (Legrand et al., 2020). A new study found that most oropharyngeal chemical stimuli are associated with polymodal receptors, particularly transient receptor potential vanilloid 1 (TRPV1) (Cui et al., 2016). Capsaicin is an agonist of TRPV1 and activates TRPV1 in peripheral sensory c-fibers. Studies have shown that TRPA1 agonist can improve swallowing by reducing laryngeal vestibular closure time, upper esophageal sphincter opening time, and penetration-aspiration score (Rofes et al., 2014).

In Tomsen's trial, patients in the capsaicin group showed a clinically relevant and statistically significant reduction in laryngeal vestibule closure (LVC) time (*p* = 0.042) (Tomsen et al., 2019). EEG revealed a highly significant correlation between the time of LVC reduction and the reduction in peak latency of pharyngeal event-related potentials. In addition, elderly patients with OD have impaired cortical responses to electrical stimulation of the pharynx compared to those without dysphagia. In elderly OD patients, the amplitudes of all peaks were significantly lower, and only the latencies of the N1 and N2 peaks were delayed (Tomsen et al., 2019). sLORETA measurement results show that patients who received the capsaicin treatment showed a significant reduction in cortical activity at the N1, P1, and N2 peaks compared with basal activation (Tomsen et al., 2019). In summary, Capsaicin can induce afferent neurons to transmit signals to the insular cortex activating feedback loops and brainstem swallowing centers through repeated repetitive stimulation of the cerebral cortex, which can restore the function of the insular cortex and induce cortical neuroplasticity, thus restoring swallowing function.

Esophageal manometry studies confirm that capsaicin exerts a prokinetic effect on esophageal motility by increasing the amplitude, duration, and speed of contraction of esophageal body peristalsis (Suntrup-Krueger et al., 2021). In Tomsen's study, there was no significant difference in the upper esophageal sphincter (*p* < 0.539). In Nakato's study, only patients who responded to capsaicin were found to have a cervical esophageal wall opening time duration was significantly shorter (*p* = 0.0003).

In Kondo's study, reflex scores for glottal closure and cough reflex improved more significantly in the capsaicin group than in the control group. Glottal closure and cough reflex are important protective mechanisms against aspiration, so capsaicin can effectively prevent aspiration pneumonia.

Our systematic review and meta-analysis had the following limitations. (1) Our meta-analysis included only 6 studies; our analysis would have been more credible if we had included more RCTs. (2) The Water Swallow Test and Swallowing Score are reliable but subjective methods for evaluating dysphagia. (3) Only English-language publications were included in our meta-analysis. (4) Heterogeneity is inevitable due to differences in gender, age, mode of administration, differences in experimental design and different disease conditions of the patients included in the studies.

## Conclusions

In summary, capsaicin may be a new perspective in treatment of oropharyngeal disorders in the elderly and stroke patients. There is still a lack of evidence for capsaicin in swallowing function, and more studies are needed to support this in the future.

## Data availability statement

The original contributions presented in the study are included in the article/Supplementary material, further inquiries can be directed to the corresponding author.

## Author contributions

CY proposed the study idea, searched the database, screened and evaluated the evidence, and wrote the manuscript. RC extracted the data and analyzed the data. MF revised the manuscript. MZ revised the English. DW, XL, and WL suggested revisions to the article. All authors read and approved the final manuscript.

## Conflict of interest

The authors declare that the research was conducted in the absence of any commercial or financial relationships that could be construed as a potential conflict of interest.

## Publisher's note

All claims expressed in this article are solely those of the authors and do not necessarily represent those of their affiliated organizations, or those of the publisher, the editors and the reviewers. Any product that may be evaluated in this article, or claim that may be made by its manufacturer, is not guaranteed or endorsed by the publisher.

## Supplementary material

Supplementary material 1 The results of this systematic review and meta-analysis registered on PROSPERO.

Supplementary material 2 Search strategy.

Supplementary Table 1 PRISMA 2020 checklist.

## Abbreviations

OD: oropharyngeal dysphagia
PSEP: pharyngeal sensory evoked potential
RCTs: Randomized controlled trials
SP: substance P
WST: water swallowing test.
