# Supplementary material for: The therapeutic effect of capsaicin on oropharyngeal dysphagia: A systematic review and meta-analysis
Source: Front Aging Neurosci. 2022 Nov 8;14:931016. doi: 10.3389/fnagi.2022.931016 (PMC9679510; doi:10.3389/fnagi.2022.931016)
Supplement: Supplementary material 1 — The results of this systematic review and meta-analysis registered on PROSPERO. [file Data_Sheet_1.PDF]

Systematic review

This record cannot be edited because it has been marked as out of scope

1. * Review title. [1 change]

Give the title of the review in English

The therapeutic effect of capsaicin on oropharyngeal dysphagia: a systematic review and meta-analysis

2. Original language title.

For reviews in languages other than English, give the title in the original language. This will be displayed with the English language title.

3. * Anticipated or actual start date.

Give the date the systematic review started or is expected to start.

25/03/2022

4. * Anticipated completion date. [1 change]

Give the date by which the review is expected to be completed.

01/12/2022

5. * Stage of review at time of this submission.

This field uses answers to initial screening questions. It cannot be edited until after registration.

Tick the boxes to show which review tasks have been started and which have been completed.

Update this field each time any amendments are made to a published record.

The review has not yet started: No

Review stage	Started	Completed
Preliminary searches	Yes	No
Piloting of the study selection process	Yes	No
Formal screening of search results against eligibility criteria	No	No
Data extraction	No	No
Risk of bias (quality) assessment	No	No
Data analysis	No	No

Provide any other relevant information about the stage of the review here.

6. * Named contact.

The named contact is the guarantor for the accuracy of the information in the register record. This may be any member of the review team.

Congwen Yang

Email salutation (e.g. "Dr Smith" or "Joanne") for correspondence:

Miss Yang

7. * Named contact email.

Give the electronic email address of the named contact.

995089435@qq.com

8. Named contact address

Give the full institutional/organisational postal address for the named contact.

Weifang Medical College

9. Named contact phone number.

Give the telephone number for the named contact, including international dialling code.

8613053848265

10. * Organisational affiliation of the review.

Full title of the organisational affiliations for this review and website address if available. This field may be completed as 'None' if the review is not affiliated to any organisation.

Weifang Medical College

Organisation web address:

11. * Review team members and their organisational affiliations.

Give the personal details and the organisational affiliations of each member of the review team. Affiliation refers to groups or organisations to which review team members belong. **NOTE: email and country now MUST be entered for each person, unless you are amending a published record.**

Miss Congwen Yang. Weifang Medical College
Mr Rudong Chen. Shandong First Medical University

12. * Funding sources/sponsors.

Details of the individuals, organizations, groups, companies or other legal entities who have funded or sponsored the review.

Without sponsorship

Grant number(s)

State the funder, grant or award number and the date of award

13. * Conflicts of interest.

List actual or perceived conflicts of interest (financial or academic).

None

14. Collaborators.

Give the name and affiliation of any individuals or organisations who are working on the review but who are not listed as review team members. **NOTE: email and country must be completed for each person, unless you are amending a published record.**

15. * Review question. [1 change]

State the review question(s) clearly and precisely. It may be appropriate to break very broad questions down into a series of related more specific questions. Questions may be framed or refined using PI(E)COS or similar where relevant.

The objective of this systematic review and meta-analysis was to assess the effectiveness of capsaicin in the treatment of dysphagia.

16. * Searches. [1 change]

State the sources that will be searched (e.g. Medline). Give the search dates, and any restrictions (e.g. language or publication date). Do NOT enter the full search strategy (it may be provided as a link or attachment below.)

The databases searched for this systematic review and meta-analysis included MEDLINE, Embase, PubMed, and the Cochrane Library. The search keywords used MeSH terms which were “swallowing” OR “swallowing disorders” OR “deglutition disorders” OR “dysphagia” AND “capsaicin” OR “TRP” OR “vanilloid receptor agonist”

17. URL to search strategy.

Upload a file with your search strategy, or an example of a search strategy for a specific database, (including the keywords) in pdf or word format. In doing so you are consenting to the file being made publicly accessible. Or provide a URL or link to the strategy. Do NOT provide links to your search **results**.

https://www.crd.york.ac.uk/PROSPEROFILES/320884_STRATEGY_20220325.pdf

Alternatively, upload your search strategy to CRD in pdf format. Please note that by doing so you are consenting to the file being made publicly accessible.

Do not make this file publicly available until the review is complete

18. * Condition or domain being studied.

Give a short description of the disease, condition or healthcare domain being studied in your systematic review.

Deterioration of swallowing function is caused by multiple conditions, including stroke, aging, and sarcopenia, and leads to reduced activities of daily life and quality of life. In addition?dysphagia can cause severe complications such as aspiration pneumonia, respiratory infection and malnutrition or death.

19. * Participants/population. [1 change]

Specify the participants or populations being studied in the review. The preferred format includes details of both inclusion and exclusion criteria.

Inclusion criteria: (1) patients with dysphagia associated with aging and stroke (Elderly or stroke patients were evaluated for Oropharyngeal dysphagia by Standardized Swallowing Assessment (SSA) scales or Volume-Viscosity Swallowing Test (V-VST) or Video Fluoroscopic Swallowing (VFS)); (2) age 18 years old; 3x)cpluaintrwiteri:w(i1ll)inpgatoietasrtwiciitaltntghisuadsyeandshitddtieaifeowrmheodconnso.ceive stimulation.; (2) patient suffers from other diseases that may affect swallowing function.; (3) patients who were allergic to capsaicin.

20. * Intervention(s), exposure(s). [1 change]

Give full and clear descriptions or definitions of the interventions or the exposures to be reviewed. The preferred format includes details of both inclusion and exclusion criteria.

Inclusion criteria: (1) patients received oral or topical capsaicin stimulation. (2) may be combined with other treatments (ice stimulation, swallowing training).

21. * Comparator(s)/control. [1 change]

Where relevant, give details of the alternatives against which the intervention/exposure will be compared (e.g. another intervention or a non-exposed control group). The preferred format includes details of both inclusion and exclusion criteria.

Patients in the control group were treated with placebo or combined with other drugs same as in the experimental group. No control group was set and studies comparing capsaicin with other treatments were excluded.

22. * Types of study to be included. [1 change]

Give details of the study designs (e.g. RCT) that are eligible for inclusion in the review. The preferred format includes both inclusion and exclusion criteria. If there are no restrictions on the types of study, this should be stated.

The inclusion criteria were (1) only published RCTs were included; (2) studies with a follow-up of at least luasniodnarliearsiat:orneeviwrimariyclt, caonmialnsu(i)etsh, oasaiethsuodp, lneotrteleantootcest.ion and data was not extractable

23. Context.

Give summary details of the setting or other relevant characteristics, which help define the inclusion or exclusion criteria.

24. * Main outcome(s). [1 change]

Give the pre-specified main (most important) outcomes of the review, including details of how the outcome is defined and measured and when these measurement are made, if these are part of the review inclusion criteria.

The primary outcomes included: swallowing function score and water swallowing test. Secondary outcomes:

efficacy of capsaicin, substance P (SP) concentrations, individual latency time, the standardized low-resolution brain electromagnetic tomography software (sLORETA), and safety of the treatment.

Measures of effect

Please specify the effect measure(s) for you main outcome(s) e.g. relative risks, odds ratios, risk difference, and/or 'number needed to treat.

25. * Additional outcome(s). [1 change]

List the pre-specified additional outcomes of the review, with a similar level of detail to that required for main outcomes. Where there are no additional outcomes please state 'None' or 'Not applicable' as appropriate to the review

Adverse effects of treatment?Electroencephalogram.

Measures of effect

Please specify the effect measure(s) for you additional outcome(s) e.g. relative risks, odds ratios, risk difference, and/or 'number needed to treat.

26. * Data extraction (selection and coding).

Describe how studies will be selected for inclusion. State what data will be extracted or obtained. State how this will be done and recorded.

Only published clinical studies will be included, and the included studies will be required to contain at least one outcome. Only studies published in English will be included. Studies must have a follow-up rate of at least 80%, and at least two main patient-important outcome included. Two authors will assess eligible studies independently.

Data extraction: We will use a standard data extraction form to retrieve the relevant data from eligible articles.

The extracted data will include: authors, study location, sample size, study design, publishing date, gender,population, age, duration of follow-up, interventions, and outcomes. If necessary, we will contact the corresponding authors of the included RCTs to make sure the information was integrated and to get any missing data.

27. * Risk of bias (quality) assessment.

State which characteristics of the studies will be assessed and/or any formal risk of bias/quality assessment tools that will be used.

The modified seven-point Jadad scale will be used to evaluate the quality of the RCTs. If the score is greater than four points, the study is considered to be of high quality. For non-RCTs, study quality will be assessed by the nine-point Newcastle-Ottawa Scale (NOS). Studies achieving a score of greater than five points are considered to be of high quality. Grading of Recommendations Assessment Development and Evaluation (GRADE) system will be used to evaluate the quality of evidence grade strength of recommendations.

28. * Strategy for data synthesis.

Describe the methods you plan to use to synthesise data. This **must not be generic text** but should be **specific to your review** and describe how the proposed approach will be applied to your data. If meta-analysis is planned, describe the models to be used, methods to explore statistical heterogeneity, and

software package to be used.

Review Manager Software for Windows (Version 5.3. Copenhagen: The Nordic Cochrane Centre, The Cochrane Collaboration, 2014) will be used to perform the meta-analyses. The mean difference (MD) or standard mean difference (SMD) will be recommended to assess continuous variable outcomes with a 95% confidence interval [CI]. For dichotomous outcomes, the results will be presented as relative risks (RR) or odds ratios (OR) with a 95% CI. The Q test will be used to evaluate the heterogeneity of studies based on the values of P and I^2. When I^2?50% and P ?0.1, the fixed-effect model will be used, otherwise, we will apply the random-effects model for meta-analysis.

29. * Analysis of subgroups or subsets.

State any planned investigation of ‘subgroups’. Be clear and specific about which type of study or participant will be included in each group or covariate investigated. State the planned analytic approach.

To be determined based on the data retrieved.

30. * Type and method of review.

Select the type of review, review method and health area from the lists below.

Type of review

Cost effectiveness
No

Diagnostic
No

Epidemiologic
No

Individual patient data (IPD) meta-analysis
No

Intervention
Yes

Living systematic review
No

Meta-analysis
Yes

Methodology
No

Narrative synthesis
No

Network meta-analysis
No

Pre-clinical
No

Prevention
No

Prognostic

No

Prospective meta-analysis (PMA)
No

Review of reviews
No

Service delivery
No

Synthesis of qualitative studies
No

Systematic review
Yes

Other
No

Health area of the review

Alcohol/substance misuse/abuse
No

Blood and immune system
No

Cancer
No

Cardiovascular
No

Care of the elderly
No

Child health
No

Complementary therapies
No

COVID-19
No

Crime and justice
No

Dental
No

Digestive system
No

Ear, nose and throat
Yes

Education
No

Endocrine and metabolic disorders
No

Eye disorders
No

General interest
No

Genetics
No

Health inequalities/health equity
No

Infections and infestations
No

International development
No

Mental health and behavioural conditions
No

Musculoskeletal
No

Neurological
Yes

Nursing
No

Obstetrics and gynaecology
No

Oral health
No

Palliative care
No

Perioperative care
No

Physiotherapy
No

Pregnancy and childbirth
No

Public health (including social determinants of health)
No

Rehabilitation
No

Respiratory disorders
No

Service delivery
No

Skin disorders
No

Social care

No

Surgery
No

Tropical Medicine
No

Urological
No

Wounds, injuries and accidents
No

Violence and abuse
No

31. Language.

Select each language individually to add it to the list below, use the bin icon to remove any added in error.
English

There is not an English language summary

32. * Country.

Select the country in which the review is being carried out. For multi-national collaborations select all the countries involved.

China

33. Other registration details.

Name any other organisation where the systematic review title or protocol is registered (e.g. Campbell, or The Joanna Briggs Institute) together with any unique identification number assigned by them. If extracted data will be stored and made available through a repository such as the Systematic Review Data Repository (SRDR), details and a link should be included here. If none, leave blank.

34. Reference and/or URL for published protocol.

If the protocol for this review is published provide details (authors, title and journal details, preferably in Vancouver format)

Add web link to the published protocol.

Or, upload your published protocol here in pdf format. Note that the upload will be publicly accessible.

No I do not make this file publicly available until the review is complete

Please note that the information required in the PROSPERO registration form must be completed in full even if access to a protocol is given.

35. Dissemination plans.

Do you intend to publish the review on completion?

Yes

Give brief details of plans for communicating review findings.?

36. Keywords.

Give words or phrases that best describe the review. Separate keywords with a semicolon or new line. Keywords help PROSPERO users find your review (keywords do not appear in the public record but are included in searches). Be as specific and precise as possible. Avoid acronyms and abbreviations unless these are in wide use.

capsaicin; swallow; dysphagia; stroke

37. Details of any existing review of the same topic by the same authors.

If you are registering an update of an existing review give details of the earlier versions and include a full bibliographic reference, if available.

38. * Current review status.

Update review status when the review is completed and when it is published.New registrations must be ongoing so this field is not editable for initial submission.
Please provide anticipated publication date

Review_Ongoing

39. Any additional information.

Provide any other information relevant to the registration of this review.

40. Details of final report/publication(s) or preprints if available.

Leave empty until publication details are available OR you have a link to a preprint (NOTE: this field is not editable for initial submission). List authors, title and journal details preferably in Vancouver format.

Give the link to the published review or preprint.
