# Supplementary material for: The therapeutic effect of capsaicin on oropharyngeal dysphagia: A systematic review and meta-analysis
Source: Front Aging Neurosci. 2022 Nov 8;14:931016. doi: 10.3389/fnagi.2022.931016 (PMC9679510; doi:10.3389/fnagi.2022.931016)
Supplement: Supplementary material 2 — Search strategy. [file Data_Sheet_2.PDF]

PubMed

72

(((vanilloid receptor agonist[Title/Abstract])) OR (((capsaicin[Title/Abstract])) OR (TRP[Title/Abstract]))) AND (((((swallowing[Title/Abstract])) OR (swallowing disorders[Title/Abstract])) OR (Deglutition Disorders[Title/Abstract])) OR (dysphagia[Title/Abstract]))

Embase

121

History Save | Delete | Print view | Export | Email Combine using And Or Collapse

#3	#1 AND #2	121
#2	**capsaicin**:ab,ti OR **trp**:ab,ti OR **'vanilloid receptor agonist'**:ab,ti	50,549
#1	**swallowing**:ab,ti OR **'swallowing disorders'**:ab,ti OR **'deglutition disorders'**:ab,ti OR **dysphagia**:ab,ti	81,021

Medline

62

AB (swallowing or swallowing disorders or deglutition disorders or dysphagia) AND AB (capsaicin or TRP or vanilloid receptor agonist)

Cochrane library

65

Advanced Search

Search | Search manager | Medical terms (MeSH) | PICO search

Save this search | View/Share saved searches | Search help

Print search history

#1	(capsaicin):ti,ab,kw OR (TRP):ti,ab,kw OR (vanilloid receptor agonist):ti,ab,kw<br>(Word variations have been searched)	Limits	1988
#2	(swallowing):ti,ab,kw OR (swallowing disorders):ti,ab,kw OR (Deglutition Disorders):ti,ab,kw OR (dysphagia):ti,ab,kw<br>(Word variations have been searched)	Limits	9001
#3	#1 and #2	Limits	65

Clear all

Highlight orphan lines

Save this search | View/Share saved searches | Search help

Print search history
